# Supplementary material for: Hybrid Models and Biological Model Reduction with PyDSTool
Source: PLoS Comput Biol. 2012 Aug 9;8(8):e1002628. doi: 10.1371/journal.pcbi.1002628 (PMC3415397; doi:10.1371/journal.pcbi.1002628)
Supplement: Text S4 — Complete source code for the PyDSTool package (version 0.88.120504). Includes API documentation and help files linking to web pages. This file is identical to the current public release on Sourceforge.net. (ZIP) [file pcbi.1002628.s004.zip › PyDSTool/html/PyDSTool.Toolbox.optimizers.criterion.criteria-pysrc.html]

xml version="1.0" encoding="ascii"?


PyDSTool.Toolbox.optimizers.criterion.criteria


| Home | Trees | Indices | Help | PyDSTool |
| --- | --- | --- | --- | --- |

Package PyDSTool ::
Package Toolbox ::
Package optimizers ::
Package criterion ::
Module criteria

[hide private]

[frames] | no frames]

# Source Code for Module PyDSTool.Toolbox.optimizers.criterion.criteria

```
  1   
  2  # Matthieu Brucher 
  3  # Last Change : 2007-08-24 14:19 
  4   
  5  """ 
  6  A list of standard convergence criteria based on the number of iterations, the last values taken by the cost function and the associated points 
  7  """ 
  8   
  9  import math 
 10  import numpy 
 11  import defaults 
 12   


13 -class IterationCriterion(object):


14    """ 
 15    A simple criterion that stops when the iteration limit is reached 
 16    """ 


17 -  def __init__(self, iterations_max):


18      """ 
 19      Initializes the criterion with a max number of iterations (iterations_max) 
 20      """ 
 21      self.iterations_max = iterations_max

 22   


23 -  def __call__(self, state, **kwargs):


24      """ 
 25      Computes the stopping criterion 
 26      """ 
 27      value = (state['iteration'] > self.iterations_max) 
 28      if value: 
 29        state['istop'] = defaults.IS_MAX_ITER_REACHED 
 30      return value

 31   


32 -class MonotonyCriterion(object):


33    """ 
 34    A simple criterion that stops when the values of the function starts to rise again 
 35    """ 


36 -  def __init__(self, ftol):


37      """ 
 38      Initializes the criterion with an error fraction for the monotony test (ftol) 
 39      """ 
 40      self.error = ftol

 41   


42 -  def __call__(self, state, **kwargs):


43      """ 
 44      Computes the stopping criterion 
 45      """ 
 46      value = (state['new_value'] > state['old_value'] * (1. + self.error)) 
 47      if value: 
 48        state['istop'] = defaults.SMALL_DELTA_F 
 49      return value

 50   


51 -class RelativeValueCriterion(object):


52    """ 
 53    The relative criterion stops the optimization when the relative error of the value is below a certain level (ftol) 
 54    """ 


55 -  def __init__(self, ftol):


56      """ 
 57      Initializes the criterion with an error fraction 
 58      """ 
 59      self.error = ftol

 60   


61 -  def __call__(self, state, **kwargs):


62      """ 
 63      Computes the stopping criterion 
 64      """ 
 65      old_value = state['old_value'] 
 66      new_value = state['new_value'] 
 67      value = (abs(new_value - old_value) / (new_value + old_value + 10e-127) < self.error) 
 68      if value: 
 69        state['istop'] = defaults.SMALL_DELTA_F_F 
 70      return value

 71   


72 -class AbsoluteValueCriterion(object):


73    """ 
 74    The absolute criterion stops the optimization when the absolute error of the value is below a certain level (ftol) 
 75    """ 


76 -  def __init__(self, ftol):


77      """ 
 78      Initializes the criterion with an error fraction 
 79      """ 
 80      self.error = ftol

 81   


82 -  def __call__(self, state, **kwargs):


83      """ 
 84      Computes the stopping criterion 
 85      """ 
 86      value = (abs(state['new_value'] - state['old_value']) < self.error) 
 87      if value: 
 88        state['istop'] = defaults.SMALL_DELTA_F 
 89      return value

 90   


91 -class RelativeParametersCriterion(object):


92    """ 
 93    The relative criterion stops the optimization when the relative error of the parameters is below a certain level (xtol) 
 94    """ 


95 -  def __init__(self, xtol, weight = None):


96      """ 
 97      Initializes the criterion with an error fraction and the weight assigned for each parameter 
 98      """ 
 99      self.error = xtol 
100      if weight != None: 
101        self.weight = weight 
102      else: 
103        self.weight = 1

104   


105 -  def __call__(self, state, **kwargs):


106      """ 
107      Computes the stopping criterion 
108      """ 
109      old_parameters = state['old_parameters'] 
110      new_parameters = state['new_parameters'] 
111      value = ((self.weight * numpy.abs(new_parameters - old_parameters) / (new_parameters + old_parameters + 10e-127)) < self.error).all() 
112      if value: 
113        state['istop'] = defaults.SMALL_DELTA_X_X 
114      return value

115   


116 -class AbsoluteParametersCriterion(object):


117    """ 
118    The absolute criterion stops the optimization when the relative error of the parameters is below a certain level (xtol) 
119    """ 


120 -  def __init__(self, xtol, weight = None):


121      """ 
122      Initializes the criterion with an error fraction and the weight assigned for each parameter 
123      """ 
124      self.error = xtol 
125      if weight != None: 
126        self.weight = weight 
127      else: 
128        self.weight = 1

129   


130 -  def __call__(self, state, **kwargs):


131      """ 
132      Computes the stopping criterion 
133      """ 
134      old_parameters = state['old_parameters'] 
135      new_parameters = state['new_parameters'] 
136      value = ((self.weight * numpy.abs(new_parameters - old_parameters)) < self.error).all() 
137      if value: 
138        state['istop'] = defaults.SMALL_DELTA_X 
139      return value

140   


141 -class GradientCriterion(object):


142    """ 
143    The gradient criterion stops the optimization when the gradient at the current point is less that a given tolerance 
144    """ 


145 -  def __init__(self, gtol, weight = None):


146      """ 
147      Initializes the criterion with an error fraction and the weight assigned for each parameter 
148      """ 
149      self.error = gtol 
150      if weight != None: 
151        self.weight = weight 
152      else: 
153        self.weight = 1

154   


155 -  def __call__(self, state, **kwargs):


156      """ 
157      Computes the stopping criterion 
158      """ 
159      gradient = state['function'].gradient(state['new_parameters']) 
160      value = ((self.weight * numpy.abs(gradient)) < self.error).all() 
161      if value: 
162        state['istop'] = defaults.SMALL_DF 
163      return value

164
```

  


| Home | Trees | Indices | Help | PyDSTool |
| --- | --- | --- | --- | --- |

|  |  |
| --- | --- |
| Generated by Epydoc 3.0.1 on Fri May 4 15:24:19 2012 | http://epydoc.sourceforge.net |
